# Extramammary Paget’s Disease of the Vulva: Report of Two Cases

**DOI:** 10.3390/medicina57101029

**Published:** 2021-09-27

**Authors:** Christoforos S. Kosmidis, Christina Sevva, Panagiota Roulia, Charilaos Koulouris, Nikolaos Varsamis, Georgios Koimtzis, Vasiliki Theodorou, Chrysi Maria Mystakidou, Eleni Georgakoudi, Georgios Anthimidis

**Affiliations:** 1 European Interbalkan Medical Center, 10 Asklipiou Street, 55535 Pylaia, Greece; dr.ckosmidis@gmail.com (C.S.K.); charilaoskoulouris@gmail.com (C.K.); nikvar83@gmail.com (N.V.); helengeorgakoudi@gmail.com (E.G.); anthimid@gmail.com (G.A.); 2 3rd Surgical Department, University General Hospital of Thessaloniki “AHEPA”, School of Medicine, Faculty of Health Sciences, Aristotle University of Thessaloniki, 1st St. Kiriakidi Street, 54621 Thessaloniki, Greece; panagiotar96@gmail.com; 3 Medical School, Faculty of Health Sciences, Aristotle University of Thessaloniki, 54124 Thessaloniki, Greece; baswtheodorou@gmail.com (V.T.); chryssa2000@gmail.com (C.M.M.); 4 Cardiff Transplant Unit, University Hospital of Wales, Cardiff and Vale University Health Board, Cardiff CF14 4XW, UK; drgxkoimtzis@gmail.com

**Keywords:** Paget’s disease, extramammary, vulva, case reports

## Abstract

Extramammary Paget’s disease is a rare condition, affecting 6.5% of all patients with Paget’s disease. The most common extramammary site is the vulvar area. Although diagnosis in some patients is difficult to set, early diagnosis is of high importance in order to detect the irreversible progression of the lesion early and prevent distant metastasis. An 89-year-old female and a 69-year-old female presented within three months with an eczematous lesion with leukoplakia in the vulva. The incisional biopsy of the skin revealed extramammary Paget’s disease. Both patients underwent a surgical wide local excision of the lesion and the specimens were sent for histopathological examination. Extramammary Paget’s disease has a high potential for distant malignancies and local recurrence, dictating that surgical excision is the most efficient treatment. The rareness of the condition and the diagnostic difficulties underline the need for early skin biopsy, which is the most efficient diagnostic tool.

## 1. Introduction

Paget’s Disease (PD) was first described in 1874 by Sir James Paget as a paraneoplastic manifestation evolving on the skin of the nipple and the areola. These lesions were characterized by an eczematous eruption of the nipple–areola complex (NAC) occurring approximately one to two years prior to mammary gland cancer [1]. However, the histopathology of the disease was not fully understood until 30 years later, when H. C. Jacobaeus suggested that, in PD, cancer cells actually originating from an preexisting neoplasm invade the epidermis of the nipple [2,3]. 

Today, and as this pathogenetic mechanism is widely accepted, PD has been observed in many extramammary sites, affecting the apocrine glands of the vulva, the pubis-groin, the perianal region, the axilla, the scrotum, and the penis [4,5,6]. While mammary PD (MPD) accounts for 0.7–4.3% of all breast carcinomas [7,8], extramammary PD (EMPD) appears even more rarely, accounting for 6.5% of all patients with PD, with vulvar PD being the most common extramammary site [8,9].

Herein, we describe two cases of vulvar PD occurring in two Caucasian elderly women and their treatment.

## 2. Case Presentations

### 2.1. Case Presentation 1

**Figure 1:**
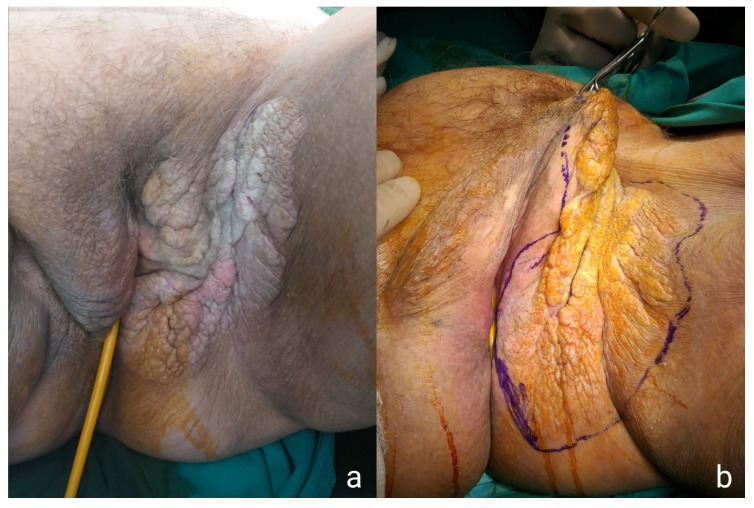
Preoperative images of the lesion showing the erythematous plaque with white patches (**a**) and the extent of the WLE (**b**).

**Figure 2:**
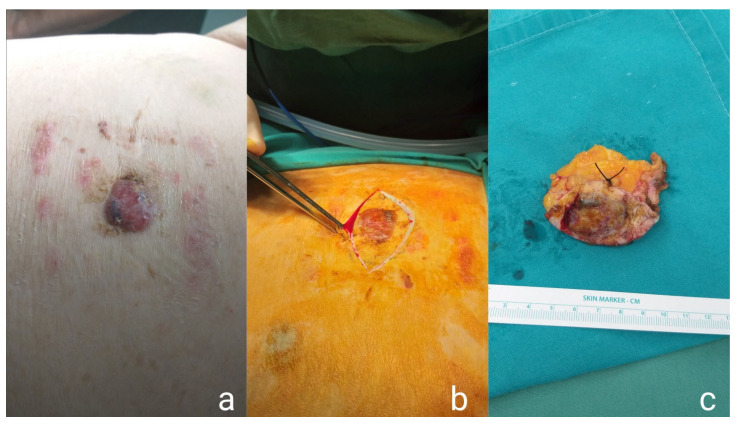
Images of the umbilical nodule upon clinical examination (**a**), the surgical excision of the nodule (**b**), and the final specimen (**c**).

An 81-year-old female presented with a large tumor-like growth covering the whole left labia majora and extending to the left inguinal area and the vagina. Upon physical examination, the lesion appeared as an erythematous plaque with white patches, slightly elevated, and with well-defined borders from the healthy tissue (Figure 1). The patient had been complaining of pruritus and an ongoing burning sensation. An umbilical nodule was also present (Figure 2a). Incisional biopsy results from the left labia majora, brought by the patient, identified the lesion as Paget’s Disease of the vulva. All cancer markers—specifically, alpha-fetoprotein (AFP), carcinoembryonic antigen (CEA), CA 19.9, CA 125, and CA 15.3—were within normal range. Gynecological examinations, including a pelvic examination, Pap smear test, and transvaginal ultrasound, were also normal. Finally, abdominal magnetic resonance imaging (MRI) was ordered, in order to reveal any perianal extension. The patient had a history of diabetes and chronic kidney disease (CKD) under hemodialysis twice a week.

**Figure 3:**
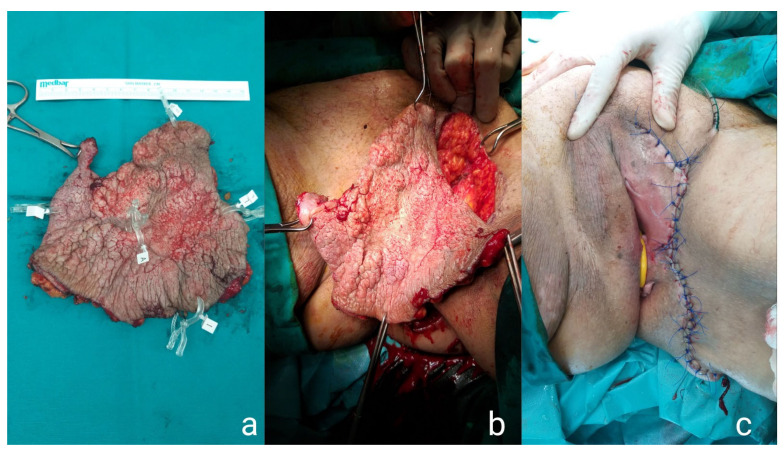
Intraoperative images of the first specimen of the WLE (**a**,**b**) and the final plastic reconstruction of the skin (**c**).

A wide local excision (WLE) of the lesion was scheduled. Under general anesthesia, the lesion was removed with a margin of 4 mm of healthy tissue. Mobilization of lateral flaps was required and a layered repair of the wound was performed (Figure 3). The patient tolerated the procedure well and underwent hemodialysis on the first day postoperatively, upon the request of the nephrology consult. The specimen comprising the left labia majora and part of the left inguinal skin measured 12.5 cm × 14.5 cm. Hyperkeratosis, hypergranulation, and thorns were present in the epidermis, and the neoplastic cells typical for PD invading all layers of the epidermis showed notable nuclear atypia. The umbilical nodule was also excised (Figure 2b,c) and histopathological examination revealed porocarcinoma, classified as pT2NX based on the 2017 annual report of the Union for International Cancer Control (UICC/2017), while negative surgical margins were achieved. The patient’s postoperative course was uneventful and the patient was discharged postoperatively on day five.

**Figure 4:**
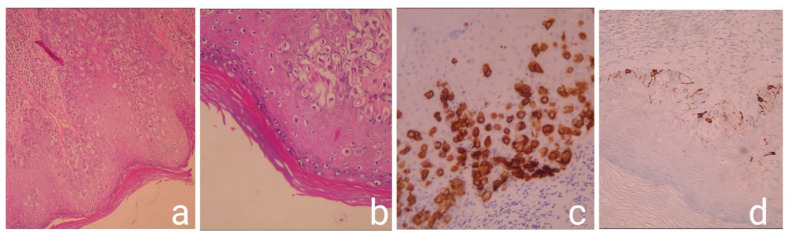
Histopathological and Immunohistochemistry images: Intraepidermal atypical epithelioid cells H&E × 100 (**a**); intraepidermal atypical epithelioid cells with moderate to severe nuclear atypia H&E × 200 (**b**); the tumor cells are positive for CK7 immunostaining × 200 (**c**); the tumor cells are negative for cocktail HMB45/melanA. Positivity is observed only in normal melanocytes immunostained × 200 (**d**).

In histopathological examination, many intraepidermal atypical epithelioid cells were observed. Immunohistochemically, the tumor cells were positive for CK AE1/AE3 and cytokeratin 7 (CK7) and negative for cocktail HMB45/MelanA, which is frequently used in the differential diagnosis of granular cell tumors and malignant melanoma. AE1 and AE3 are monoclonal antikeratin antibodies also frequently used for differential staining (AE1 stains the basal cell layer in normal human epidermis and AE3 stains the whole epidermis). Therefore, the diagnosis of Paget’s disease was confirmed (Figure 4).

### 2.2. Case Presentation 2

**Figure 5:**
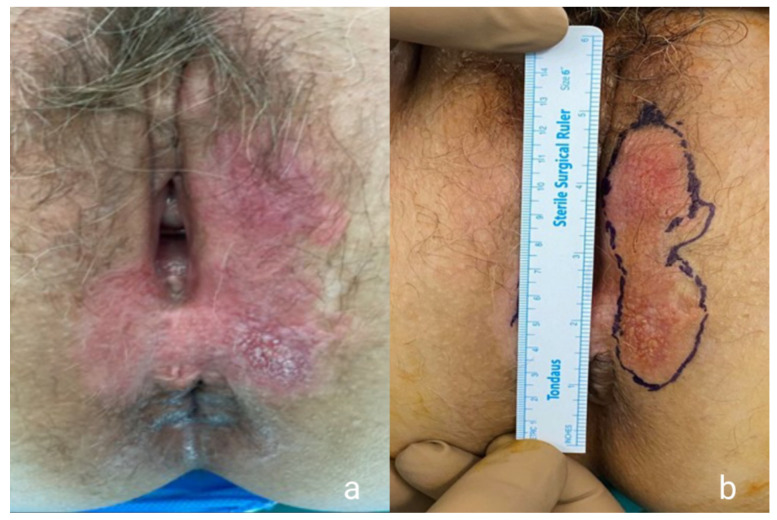
Preoperative images showing the typical white plaque and the erosions (**a**) and the extent of the WLE (**b**).

A 69-year-old female presented with symptoms of pruritus and swelling in the area of the vulva and the perineum. Clinical examination revealed a large eczematous plaque with a typical white scale and erosions extending from the skin of the left labia majora to the perianal area (Figure 5). A second, smaller skin lesion of the lower part of the right labia majora was also observed. The same diagnostic routine was also followed for this patient. Gynecological examination and cancer markers appeared normal, whereas the MRI that was obtained confirmed the perianal extension. All other laboratory results (i.e., complete blood count test and chemistry panel) were normal. Incisional results from the left labia majora identified the lesion as EMPD located at the area of the vulva. The patient also had a medical history of hypertension, dyslipidemia, and atrial fibrillation, for which she was receiving acenocoumarol.

**Figure 6:**
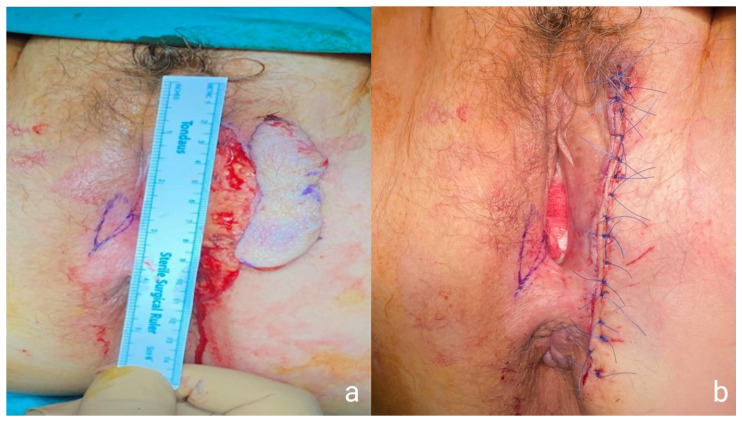
Intraoperative images of the second patient’s WLE (**a**) and the final plastic reconstruction of the skin (**b**).

**Figure 7:**
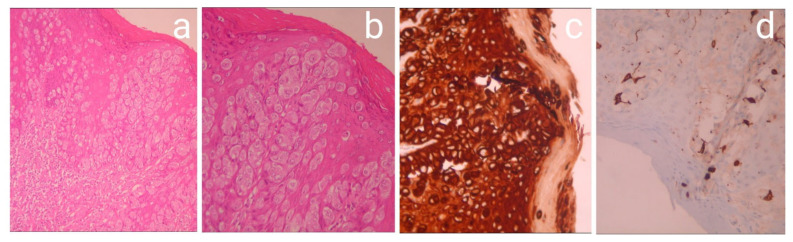
Intraepidermal atypical epithelioid cells H&H × 100 (**a**); intraepidermal atypical epithelioid cells with moderate to severe nuclear atypia H&H × 200; (**b**) the tumor cells, positive for CK AE1/AE3 immunostaining × 200 (**c**); the tumor cells, negative for cocktail HMB45/melanA. Positivity is observed only in normal melanocytes immunostained × 200 (**d**).

The patient underwent surgical WLE of the lesion, as well, under general anesthesia. A skin-subcutaneous resection was performed, followed by a plastic reconstruction of the skin (Figure 6). Skin flaps were mobilized bilaterally, and tissue layers were sutured. The patient did not face any post-operative morbidity and was discharged with medical instructions after 24 h of hospitalization. The results of the histopathological examination verified the presence of EMPD. The two specimens from, the left and right labia majora, measured 2.4 cm × 7.8 cm and 1 cm × 2 cm, respectively. In immunohistochemistry analysis, neoplastic cells positive in the keratin examination AE1/AE3 were present in a small part of the specimen. The surgical margins of the specimen were free of neoplastic cells (Figure 7).

## 3. Discussion

Although the exact prevalence of EMPD is not known, most authors who have run retrospective studies for EMPD patients conclude that the condition affects mostly white women, with a mean age of 72 ± 4 years old [4,9,10,11,12,13,14]. Depending on the existence of a synchronous or non-synchronous underlying adenocarcinoma or distant tumors, EMPD is often classified as “primary” or “secondary”. “Primary”, or “intraepithelial”, EMPD refers to cases that cannot be linked to another malignancy, whereas “secondary” EMPD is used when underlying malignancies are present [4,10,12].

In a retrospective study of vulvar EMPD, Shaco-Levy et al. studied the exact location of the lesions. The most common sites, accounting for 68% of the patients, were the major labia followed by the minor labia [13].

The clinical presentation does not show any differences between those subgroups, as the classification refers only to the detection of another cancer. Usually, patients complain of pruritus, burning sensations, and pain. In areas with a high concentration of apocrine glands, oedema, erythematous lesions, leukoplakia, hyperkeratosis, and sometimes ulcerations and bleeding can be observed [6,8,9,10,13]. However, EMPD may have an atypical presentation, as many patients are asymptomatic and quite often misdiagnosed. Other dermatological conditions mainly affecting post-menopausal women, such as dermatitis, lichen sclerosus, fungal infection, vulvovaginitis, or intertriginous dermatitis [13,15], are used as the primary diagnosis, leading to a delayed EMPD diagnosis, usually within one to two years following the first symptoms’ appearance [4,12,13,14]. The differential diagnosis also includes inverse psoriasis, dermatophytosis, condyloma accuminata, mycosis fungoides, candidiasis, histiocytosis, Bowen’s disease, atrophy, ulcer, anogenital intraepithelial neoplasm, basal cell carcinoma (although highly improbable due to macroscopic and dermoscopic patterns), or squamous cell carcinoma or melanoma [4,15].

Most retrospective studies, including a large meta-analysis by Snast et al. [16], indicate that EMPD seems to affect women more often, probably as a result of the condition’s predominance in the vulvar area [4,6,8,10,12]. However, in one retrospective study of 76 Japanese patients, Hatta et al. have found a significantly higher prevalence in men; in this particular study, 72% were male and 28% female [14]. Shabihkhani et al. have also published an analysis of 20 cases of EMPD of the scrotum [5].

Another important difference between MPD and EMPD derives from immunohistochemistry analysis. Several antibodies with specificity to various types of mucin core proteins that are not expressed in healthy tissue have been reported as useful tools in order to distinguish between EMPD and MPD. The most commonly found and widely accepted one is the expression of Mucin 5AC (MUC5AC)—a gastric-type, secretory mucin—only in EMPD specimens and especially in the primary type [15,17], as Yoshii et al. indicated in a series of 36 cases in 2002, where all 36 (100%) tested positive for MUC5AC [15]. Since then, Fernandez-Florez et al., after analyzing five patients [11] have reported the expression of MUC5AC in all of them. Recently, Hata et al. suggested that MUC5AC is associated with invasive EMPD. In this immunohistochemical analysis, 43.2% of specimens expressed MUC5AC [18].

Although promising, this marker has limitations, and its use is not yet generally accepted as a standard procedure in clinical practice. More data, deriving from larger studies, are required in order for immunohistochemistry to be established as a diagnostic and prognostic tool for EMPD. The definitive diagnosis of EMPD is set through histopathology. A skin biopsy of the lesion is the gold standard, followed by hematoxylin and eosin stains together with the evaluation of cytokeratin 7 (CK7) and cytokeratin 20 (CK20) for further classification of the disease and evaluation of invasive disease [12,19,20]. Another similar practice is a mapping (scouting) biopsy, where the biopsy includes cuts from different parts of the lesion [21]. However, in order to investigate the existence of an underlying adenocarcinoma or distant tumors, a careful clinical examination of the lymph nodes and a full diagnostic workup based on the patient’s gender should be performed. This includes mammography; pelvic ultrasonography; Papanicolaou smear testing for female patients; prostate-specific antigen (PSA) blood testing for male patients; colonoscopy; cystoscopy; computed tomography (CT) of the chest, abdomen, and pelvis; and serum tumor markers (CEA, CA 19-9, CA 15-3) for both genders [8,12,14,15].

In vulvar PD, several treatment options have been considered and even implemented, some of them with ambiguous efficacy. In general, treatment can be divided into surgical and non-surgical means. Surgical treatment includes both conservative and radical procedures. Conservative procedures include wide local excisions and simple or partial vulvectomies, whereas total and radical vulvectomies are classified as radical procedures [9,13,20]. Other surgical approaches, suitable for several other skin cancers, have occasionally been implemented for vulvar EMPD, such as Mohs micrographic surgery (MMS) and linear strip skin biopsy [21]. MMS appears to be the most promising technique in terms of preserving healthy tissue. However, certain technical difficulties regarding this technique seem to put limitations on its use [20,21]. Linear strip skin biopsy (originally used for melanoma management), and its emerging variants, are repeated by taking consecutive strips of skin until histopathology results confirm that margins are negative for EMPD [21,22]. Finally, reconstruction surgeries using skin grafts and flaps are sometimes performed in the vulvar area, usually with poor results, as flaps and grafts might cause inadequate wound healing or necrosis, concealment of a possible recurrence, and even higher recurrence rates [13,21,23].

Non-surgical techniques have also been developed or used in vulvar EMPD, most of them as complementary treatments to the surgical ones. Snast et al., in their recent meta-analysis for non-surgical treatments for EMPD, have reviewed the efficiency of the options available in the literature, specifically radiotherapy, photodynamic therapy (PDT), laser therapy, immune response modifiers, and locally applied creams [16]. Radiation is mostly used in elderly patients where comorbidities do not allow surgical intervention, in combination with surgical treatments as adjuvant radiation [13], or in difficult anatomic areas where complete excision of the lesion is not feasible [16,20,21]. Laser ablation (CO_2_, holmium, argon dye) has been used on several occasions; nevertheless, high local recurrence rates have been reported and patients complain of severe pain [13,16,20,21]. In PDT, which is widely used in several dermatological conditions, a photosensitizing agent together with specific visible light wavelengths is administered to the patient. This process produces reactive oxygen species that aim to selectively destroy the tumor [20,21]. Up until now, PDT treatments include topical 5-aminolevulinic acid (ALA), topical methyl aminolevulinate (mALA), systemic photosynthesizer, and topical or systemic agents, with an overall complete response rate of 36%, suggesting that PDT probably has limited use in EMPD [16]. Other local treatments have been tested in an attempt to develop less aggressive management options. Imiquimod 3.75% and 5% creams, topical bleomycin 3.5%, 5-fluorouracil (5-FU) cream, and calcipotriene 0.005% cream (a vitamin D analogue) have shown satisfactory results with clinical improvement, but not as a monotherapy [16,20,21,24,25]. Finally, targeted therapy has been attempted to treat metastatic EMPD. As 20–60% of EMPD patients show an overexpression of the human epidermal growth factor receptor 2 (HER-2), several recombinant monoclonal antibodies against HER-2—such as trastuzumab with or without docetaxel, lapatinib, and apatinib—have been recruited against metastatic EMPD [21,26]. Although anti-HER-2 therapy has been tried in very few patients, it seems to reduce tumor size, and it is well tolerated by the patients. Very recently, trastuzumab-based treatments, mostly a paclitaxel–trastuzumab combination, have been described as leading to a complete or partial response in patients with invasive vulvar PD, thus strongly indicating the possibility that HER-2 inhibitors are suitable for adjuvant therapy [27].

All these non-surgical treatments seem promising in offering less-aggressive alternative treatments; however, future studies are needed to further investigate their efficacy. Nevertheless, the gold standard remains surgical excision combined with regular follow-ups. In both our cases, wide surgical excision with the removal of the skin and subcutaneous tissue was the treatment of choice. Later on, histopathology revealed that the surgical margins of both specimens were clear of the neoplasm. On many occasions in the literature, negative margins appear to be difficult to achieve, due to the expansion of the lesion to the vagina. However, several studies have shown that there is no statistically significant correlation between negative surgical margins and lower overall survival or recurrence rates [9,13,14].

Regular follow-up is of high importance for patients with vulvar EMPD, as EMPD can relapse up to 15 years after being first diagnosed [19]. Official guidelines for EMPD post-surgical management, in terms of frequency and the kind of diagnostic workup, are not available. The authors, based on the literature, will approach the patients by conducting a thorough clinical examination of the initially affected area, evaluating lymph nodes, and assessing imaging and laboratory results every three months for the first three years, then twice a year for the following two years. After that, a clinical evaluation will be done annually, in order to detect a possible local recurrence or distant metastasis early. Laboratory tests will include serum levels of tumor markers (CEA, CA19-9, CA 15-3), and imaging means will consist of thoracic, abdominal, and pelvic CT scans.

## 4. Conclusions

Vulvar EMPD is a rare condition with atypical clinical presentation. It is crucial for the patient’s overall survival to overcome the diagnostic challenges mentioned above and proceed rapidly to the performance of biopsy, thus avoiding perseverance on other benign dermatological conditions. High clinical suspicion in septuagenarian and octogenarian women with eczematous lesions and leukoplakia is the key to an early diagnosis.

All patients baring a biopsy where PD cells are present should be treated with wide local excision, while other non-surgical treatments can be considered depending on the patient’s individual characteristics and the histopathology results. Frequent and thorough follow-up is of great importance, as EMPD is usually followed by metastatic tumors and has a high rate of local recurrence.
